# Lead Poisoning and Anemia Associated with Use of Ayurvedic Medications Purchased on the Internet — Wisconsin, 2015

**DOI:** 10.15585/mmwr.mm6432a6

**Published:** 2015-08-21

**Authors:** Jon Meiman, Robert Thiboldeaux, Henry Anderson

**Affiliations:** 1 Epidemic Intelligence Service, CDC; 2 Wisconsin Division of Public Health, Bureau of Environmental and Occupational Health

On April 30, 2015, the Wisconsin Division of Public Health (WDPH) was notified by a local health department of an elevated blood lead level (BLL) in a female patient aged 64 years. All Wisconsin laboratories are required to provide BLL testing results performed on any state resident to WDPH, and WDPH and local health departments are statutorily mandated to investigate any single BLL ≥20 *μ*g/dL or BLLs that are persistently ≥15 *μ*g/dL. Review of medical records revealed that the patient had developed progressive fatigue and shortness of breath during a period of multiple weeks that prompted inpatient medical evaluation. Hemoglobin level was 8.3 g/dL (normal range for age and sex of patient = 12.5–15.0 g/dL), and peripheral blood smear showed normochromic, normocytic red blood cells with basophilic stippling. A BLL was obtained and found to be 85.8 *μ*g/dL. Urine toxic metals tests revealed mercury and aluminum levels in the normal range. Combined methylated and inorganic urine arsenic levels were slightly elevated at 53.3 *μ*g/L (normal = <18.9 *μ*g/L). The patient was discharged for outpatient lead chelation therapy with oral meso-2,3-dimercaptosuccinic acid.

WDPH interviewed the patient to determine possible environmental sources of lead. She did not report any home remodeling that involved paint disturbance or plumbing maintenance, symptoms consistent with pica, use of pottery manufactured outside the United States, or ingestion of wild game, which can contain lead shot fragments (1). She reported taking several supplements, including two Ayurvedic (traditional Indian) medications produced in India that she purchased on the Internet: Mahayogaraj Guggulu (MG) (Sri Sri Ayurveda Trust) and Bruhat Vata Chintamani Rasa (BVCR) (Shree Dhootapapeshwar Limited). The patient ingested approximately four tablets of MG and two tablets of BVCR daily during February–April 2015.

The Wisconsin State Laboratory of Hygiene performed metals testing of the patient’s well water using graphite furnace atomic absorption, and of both Ayurvedic medications using inductively coupled plasma optical emission spectroscopy. Well water lead level was 4.3 *μ*g/L (Wisconsin public health standards set acceptable levels at ≤15 *μ*g/L), and arsenic was undetectable. BVCR contained 16.4 mg/kg (0.2%) lead, and MG contained 48,700 mg/kg (4.9%) lead. Both supplements also contained trace amounts of cadmium, chromium, and aluminum, as well as substantial amounts of arsenic (3,830 mg/kg in MG) and thallium (14.7 mg/kg in MG and 17.2 mg/kg in BVCR). On the basis of estimated daily MG and BVCR consumption and the patient’s body weight, the patient’s exposure to arsenic and thallium exceeded thresholds deemed safe for human health, as defined by the U.S. Environmental Protection Agency (2). The patient discontinued Ayurvedic medication use and reported improvement in symptoms after 1 month of chelation therapy.

Lead is a highly toxic substance that has no endogenous physiologic role, and no safe level of exposure has been identified. High levels of exposure can cause anemia, cognitive dysfunction, coma, and death (3). Although strict regulations have substantially reduced environmental contamination in the United States, lead poisoning continues to occur. This case report confirms earlier reported risk for lead poisoning from Ayurvedic medications produced in India (4), and highlights the acute toxicity that can develop from short-term use. Although toxic metals can occur naturally in some Ayurvedic medicines, or result from contamination, metals such as lead are often intentionally added to some preparations because of putative health benefits (e.g., *naga bhasma*, a lead-based herbal medicine used to treat various conditions). Physicians should be aware of possible toxicity caused by these medications and should consider lead poisoning as a cause of unexplained anemia in patients taking Ayurvedic medication. Although this investigation did not reveal health problems caused by other toxic metals, the elevated levels of arsenic and thallium could have presented health risks if these medications had been consumed for prolonged periods. State and local public health departments should consider outreach to educate the public about potential risks of Ayurvedic medications and consider sales restrictions as permitted by statutory and regulatory authority.
